# COLGALT1 is a potential biomarker for predicting prognosis and immune responses for kidney renal clear cell carcinoma and its mechanisms of ceRNA networks

**DOI:** 10.1186/s40001-022-00745-5

**Published:** 2022-07-16

**Authors:** Shiwei Liu, Yang Yu, Yi Wang, Bingye Zhu, Bangmin Han

**Affiliations:** 1 Department of Urology, Shanghai General Hospital of Nanjing Medical University, Shanghai, 200080 China; 2 Department of Urology, Affiliated Hospital of Nantong University, Jiangsu Province, Nantong, 226001 China; 3 Department of Urology, Shanghai General Hospital, Shanghai Jiao Tong University School of Medicine, Shanghai, 200080 China; 4 Department of Urology, Affiliated Nantong Hospital of Shanghai University, The Sixth People’s Hospital of Nantong), Jiangsu Province, Nantong, 226001 China

**Keywords:** COLGALT1, Kidney renal clear cell carcinoma, Immunity, Prognosis, ceRNA

## Abstract

**Background:**

As precision medicine gradually played an inaccessible role in cancer treatment, there was an urgent need to explore biomarkers or signatures for predicting cancer prognosis. Currently, little was known about the associations between COLGALT1 and kidney renal clear cell carcinoma (KIRC). Hence, this study was performed to reveal its roles in KIRC and to identify potential mechanisms of competing endogenous RNA (ceRNA) networks.

**Methods:**

R 4.1.1 software was utilized to conduct bioinformatics analyses with the data derived from online databases. Difference analysis, survival analysis, univariate/multivariate cox regression analysis and correlation analysis were carried out successively in this article. Besides, we also investigated potential effects and mechanisms of COLGALT1 in KIRC.

**Results:**

COLGALT1 expression was overexpressed in KIRC samples compared with the normal samples and it was associated with poor OS (*P* < 0.001). COLGALT1 was also found to be significantly related to clinicopathological characteristics such as grade, T, N, M, stage and Cox regression analysis with univariate and multivariate data suggested it might be an independent prognostic parameter in KIRC (*P* < 0.001). Furthermore, Seven significantly enriched pathways were identified. Interestingly, correlation analyses revealed an association between COLGALT1 and microsatellite instability (MSI), tumor mutational burden (TMB) and immunity (*P* < 0.001). In addition, we used TIDE and TCIA databases to predict the immune response of COLGALT1 in KIRC and it suggested low expression of COLGALT1 is more likely to benefit from immunotherapy. Besides, we identified a ceRNA network of SLC16A1-AS1/hsa-mir-502-3p/COLGALT1 for its potential mechanism. Finally, experiments in vitro indicated that COLGALT1 was significantly related to cell proliferation.

**Conclusions:**

COLGALT1 could act as a valid immune-related prognostic indicator for KIRC and participated in a ceRNA network of SLC16A1-AS1/hsa-mir-502-3p/COLGALT1, offering one potential biomarker to investigate the mechanism and clinical therapeutic value of KIRC.

**Supplementary Information:**

The online version contains supplementary material available at 10.1186/s40001-022-00745-5.

## Introduction

Globally, renal cell carcinoma ranks among the top ten cancers in both males and females [1]. The main histology of renal cell carcinoma, kidney renal clear cell carcinoma (KIRC), is the most lethal type, which approximately accounts for 75% of all cases, and the 5-year survival rate is very low [2]. Despite the fact that 5-year survival rate for KIRC has improved significantly in recent decades, the prognosis for those diagnosed with advanced stages is still poor [3]. Hence, it is crucial to find biomarkers as prognostic indicators as well as evaluate its potential clinical therapeutic value of KIRC.

Post-translational modifications (PTMs) are commonly found to be associated with cancer cells, and substantial evidence indicates that PTM plays a key role in regulating the tumorigenesis, progression, and metastasis of malignant tumors [4, 5]. Glycosylation modifications are the most common yet complex post-translational modifications, which have a profound impact on protein function, stability, subcellular localization and other traits [6]. Relevant studies suggested that dysregulated glycosylation serves as a crucial role in tumor progression and development [7, 8]. As one type of glycosylation modification, glycosylation of collagen is originated from the endoplasmic reticulum through the galactosyltransferases collagen beta(1-O)galactosyltransferase 1 (COLGALT1) and COLGALT2 [9]. Several studies have reported that the mutations of COLGALT1 could cause abnormalities to the brain's small blood vessels and perforated brain malformations [10]. More importantly, related research indicated that the collagen glycosylation could regulate cell adhesion and spreading on basement membranes which could play a critical step in the process of metastatic tumor [11].

As the same manner described in our previous article [12], we finally mined COLGALT1 as a potential biomarker in TCGA–KIRC data set, after a series of gene filtration methods. In this study, we carried out several bioinformatic analyses by website tools and R scripts to assess the clinicobiological role and immunotherapeutic value of COLGALT1 in KIRC. Moreover, several experiments in vitro were conducted to explore potential effects of COLGALT1 in KIRC cell lines. This current research could provide a unique perspective to predict the value of prognosis and evaluate the potential clinical therapeutic of COLGALT1 in KIRC.

## Materials and methods

### Raw data collection and patient samples

We obtained data for a total of 72 normal and 539 KIRC samples from TCGA database, including its raw RNA-seq reads with the corresponding clinical data [13]. We applied the R package Bioconductor for processing and normalizing the COLGALT1 expression in samples obtained from the database, and further analyses were performed using these data. The tissues of KIRC samples and corresponding pericarcinous samples were obtained from patients undergoing KIRC surgery in our institution. Collected tissues were fixed in 4% paraformaldehyde and stored at −80 °C for further study. The current study obtained the approval of the Institutional Review Board in Shanghai General Hospital, and all patients signed an informed consent form.

### Differentially expression analysis

Through R software (https://www.r-project.org/), we analyzed the raw data from the TCGA. The expression levels of mRNA, miRNA and LncRNA in KIRC were calculated with “limma” packages in R [14]. The cutoff standard of different expression genes (DEGs) was determined as |log2FC (log2fold-change)|> 1 and adjusted *P* value (*P*) < 0.05, and COLGALT1 was subsequently identified. In addition, we verified the COLGALT1 expression in other public databases including GEO (Gene Expression Omnibus; GSE6344) and ICGC (International Cancer Genome Consortium). Then, the protein expression of COLGALT1 was obtained through the CPTAC database (clinical proteomic tumor analysis consortium) for KIRC [15]. Next, the correlations between COLGALT1 expression and clinicopathological characteristics were investigated based on above databases through R language. Kruskal–Wallis or Wilcoxon rank sum test was served as the significance test by virtue of the clinical stages for comparison. HPA (Human Protein Atlas, http://www.proteinatlas.org) as one public platform provides representative immunohistochemical protein expression data of common cancers [16]. In this study, immunohistochemical images of protein expressions of COLGALT1 between KIRC and normal samples were directly visualized by HPA.

### Cell culture and Transfection

Renal carcinoma cell line Caki-1 and Human renal tubular epithelial cell Hk-2 were ordered at the Shanghai Enzyme-linked Biotechnology Co., Ltd. Caki-1 and Hk-2 were maintained in DMEM and DMEM/F12 medium, respectively, with 10% FBS and 1% penicillin/streptomycin at 37 °C with 5% CO2. KIRC cell line Caki-1 was transfected with negative control (NC) vector and vector of COLGALT1 using lipo3000 reagent (Invitrogen). The vectors of COLGALT1 knockdown and corresponding negative control (NC) were ordered at Gene Pharma. We cultured Caki-1 cells in 6-well plates and used 3ug of plasmid per well for transfection. We conducted further researches after 48 h of cell incubation.

### CCK-8 assay

CCK-8 (Cell Counting Kit-8) was performed to evaluate the proliferation of cells. CAKI-1 cells transfected with the negative control and COLGALT1 knockdown vector were transferred with a density of 1000 cells per well in a 96-well plate. After culturing for 24 h, 100 μL serum-free DMEM medium was added into each well, which containing 10% CCK-8 reagent. After incubation for 2 h at 37 °C, we used microplate spectrophotometer to measure the 450 nm OD value in each well.

### qRT-PCR

Total RNA of both tissues and cells was extracted by TRIzol (Takara, Japan). Through ReverTra Ace qPCR RT Kit (Toyobo, China), we conduct the reverse transcription reaction using extracted RNA (1 µg). Each reaction of the qRT-PCR was performed using the resulting cDNAs (1 µl) using iQTM SYBR^®^ Green Supermix (Bio-Rad, USA) with a final 10 µl volumes. GAPDH was utilized to standardize the mRNA expression, and the data were expressed as an expression of fold change through the 2^−ΔΔCt^ method. COLGALT1 primer: 5′-ACTCCACGGAATGGTACAAAC-3′ (forward); 5′-CTACGGACCACAACATGGATAAC-3′ (reverse).

### Western blotting

After transfection, cells were isolated using RIPA buffer with 1/100 PMSF. Through SDS–PAGE gel (10%), loaded proteins were separated and further transferred to PVDF membranes. The membrane was then incubated using primary antibodies including COLGALT1 (Proteintech) and ACTB (Abcam) after blocking with 5% BSA at 4 °C overnight, then incubated in peroxidase-conjugated secondary antibody (CST) at room temperature for 1 h. Through the FluorChem M system (Protein Simple), immunopositively bands were visualized with an enhanced chemiluminescence kit. We further used the Image J software to conduct the quantification of western blot images.

### Functional and pathway enrichment analysis

KEGG serves as an commonly encyclopedia of genomes utilized for pathway enrichment analysis [17]. By means of gene set enrichment analysis (GSEA), target genes were analyzed using the database to annotate, visualize and integrate discovery to find potential pathways (http://www.kegg.jp/). Nominal FDR less than 25% and *P* value (*P*) below 0.05 were deemed as statistically significant.

### Evaluation of tumor mutational burden, microsatellite instability and neoantigen

Based on the Sangerbox website (http://sangerbox.com/), the correlations between COLGALT1 and MSI (microsatellite instability), TMB (tumour mutation burden), neoantigen were calculated. The MSI and TMB were defined as numbers of deletion or insertion event which occurred in repetitive gene sequences and the overall mutation rates per million base pair, respectively [18].

### Analysis of immune cell infiltration and tumour microenvironment

ESTIMATE is one method used to predict the proportion of immune and stromal cells in tumour samples based on the gene expression signatures [19]. We utilize this tool to assess the correlation between COLGALT1 expression and the components of tumour microenvironment in KIRC. Meanwhile, TIMER [19] is another Immune Estimation Resource website utilized to assess the relationship between COLGALT1 expression and immune cell infiltration such as CD8+T cell, B cells, dendritic cells, CD4+T cells, neutrophils and macrophages in KIRC.

### Prediction of immune response of COLGALT1 to immunotherapy

TIDE (Tumor Immune Dysfunction and Exclusion) and TCIA (The Cancer Immunome Atlas) databases were utilized to predict the response of COLGALT1 to immunotherapy. The correlation analysis between COLGALT1 expression and drug sensitivity was performed using CellMiner data set (http://discover.nci.nih.gov/cellminer/). Data processing and graphing were carried out through R/Bioconductor package of the “ggpubr”, “limma” and “impute”.

### Statistical analysis

All statistical data analysis and figures were carried out on SPSS 24.0 (IBM, Chicago, USA), GraphPad Prism 9.0 (San Diego, USA) and R 4.1.1 (https://www.r-project.org/). Correlation analysis between genes was carried out with Pearson’s correlation method. Prognostic analysis of COLGALT1 was carried out by log-rank test and Kaplan-Meier curve. Cox regression analysis including univariate and multivariate was applied to evaluate the relationships between variables and OS. The STRING and Cytoscape software were utilized for the construction and visualization of PPI (protein-protein interaction) network, respectively. The Wilcoxon test was utilized to calculate TIDE score between groups. All statistical data with *p* values below 0.05 were thought to be statistically significant.

## Results

### COLGALT1 expression in KIRC

**Fig. 1:**
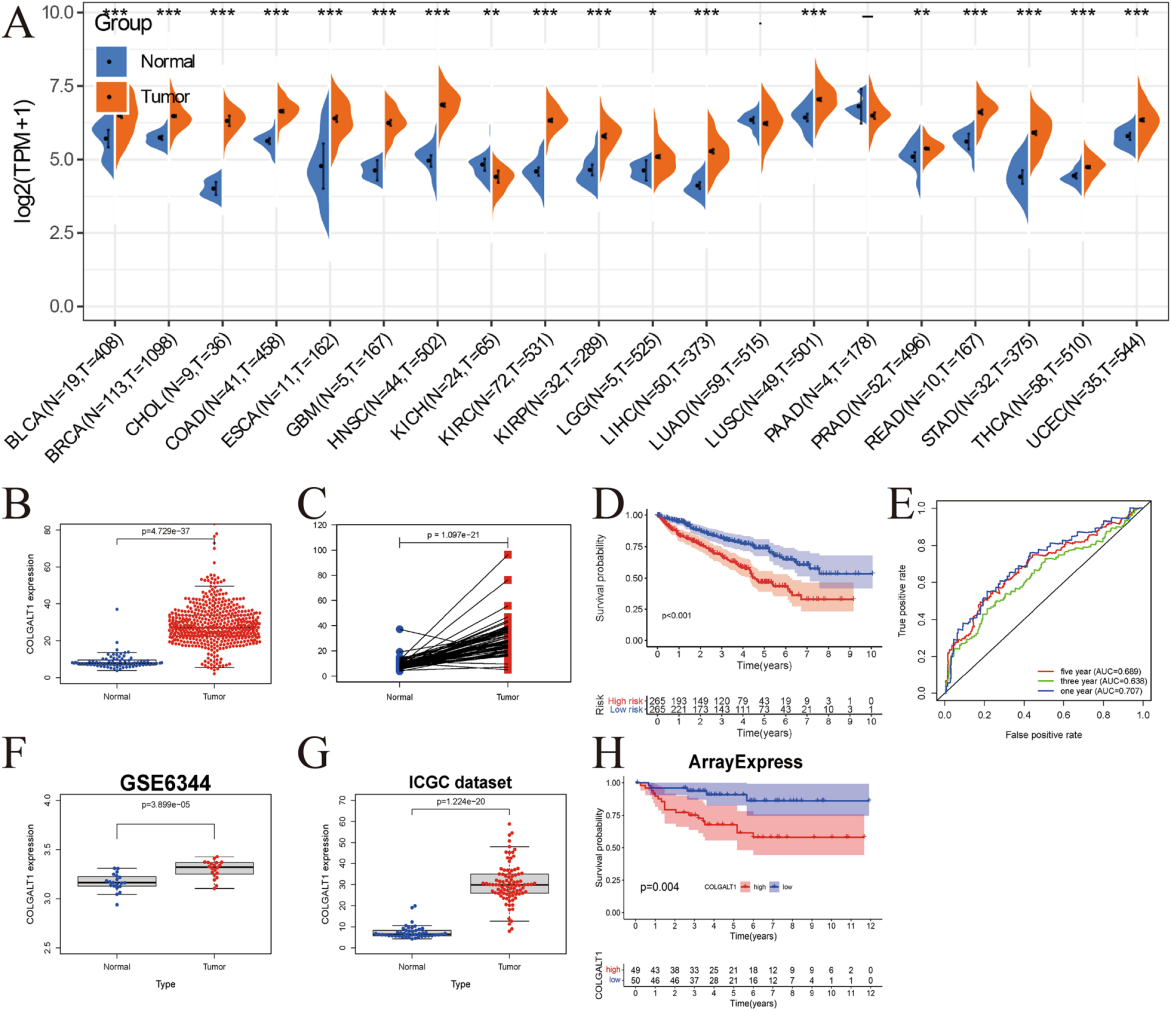
**A** Pan-cancer analysis of the expression of COLGALT1 in TCGA database; **B** expression of COLGALT1 in KIRC samples and normal samples in TCGA data set; **C** expression levels of the COLGALT1 expression between KIRC samples and their paired normal samples in TCGA data set. **D** Overall survival curve of COLGALT1 in TCGA–KIRC data set. **E** ROC curves of COLGALT1 in TCGA–KIRC data set; **F**, **G** Expression of COLGALT1 in KIRC samples and normal samples in GEO and ICGC data sets. **H** Overall survival curve of COLGALT1 in ArrayExpress data set. *P < 0.05; **P < 0 .01; ***P < 0 .001

Using pancancer sequencing data from the TCGA database, we evaluated the expression of COLGALT1 in various tumour samples compared with normal samples. We noticed COLGALT1 is highly expressed in cholangio carcinoma (CHOL), glioblastoma (GBM), bladder urothelial carcinoma (BLCA), KIRC, etc. and down-regulated in Kidney Chromophobe (KICH) compared with corresponding normal samples from the TCGA data set (Fig. 1A, B). The expression of COLGALT1 was then compared in 72 pairs of samples including KIRC tissue and the para-cancerous tissue, and we found COLGALT1 is similarly up-regulated in tumour tissues (Fig. 1C). The medium expression of GOLGAT1 was defined as the cutoff point, and then patients with KIRC were classified into low-risk and high-risk groups. These two groups of KIRC patients predicted significantly different survival curves, and higher COLGALT1-expression patients tended to have a worse prognosis. (*P* < 0.001, Fig. 1D). To assess the prognostic capability of the COLGALT1 signature, the area under the ROC curve was evaluated. In addition, the AUC was 0.707, 0.638, and 0.689 for patients' 1-year, 3-year, and 5-year survival, respectively (Fig. 1E). Besides, through the ICGC and GEO databases (GSE6344), we found that COLGALT1 is also up-regulated in kidney tumor tissues (Fig. 1F, G). High expression of COGALT1 was also associated with a worse outcome than a low level of expression in the ArrayExpress data set (*P* = 0.004, Fig. 1H).

**Fig. 2:**
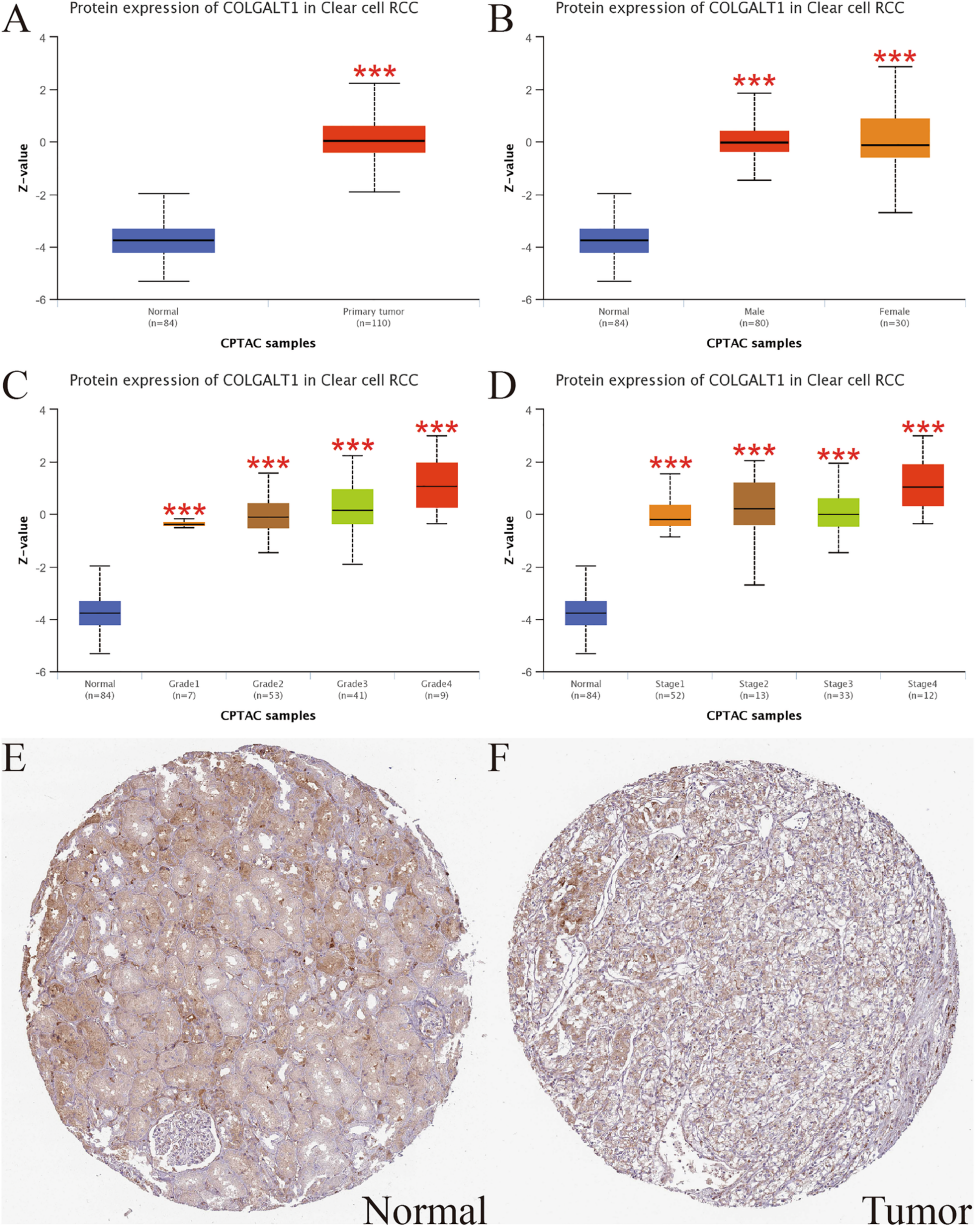
Associations between COLGALT protein expression and clinicopathologic characteristics, including **A** primary tumor, **B** gender, **C** grade, **D** stages. **E**, **F** Immunohistochemical pictures from the HPA database indicates that COLGALT1 is highly expressed in tumor tissues. ***P < 0 .001

**Fig. 3:**
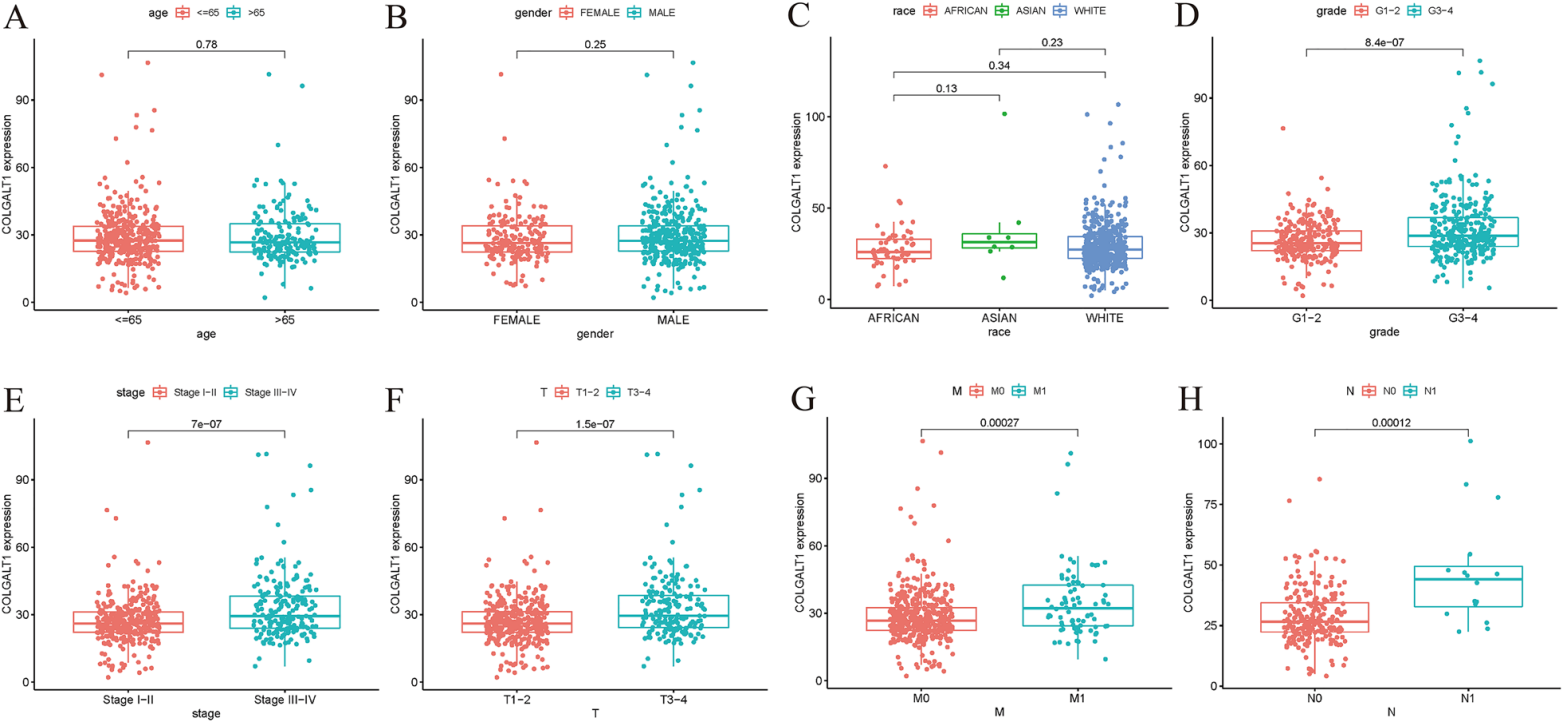
Associations between COLGALT expression and clinicopathologic characteristics, including **A** age, **B** gender, **C** race, **D** grade, **E** stage, **F**–**H** T, N, M stage

We further investigated the connection between COLGALT1 protein expression and different clinicopathological characteristics through CPTAC database. We found high protein expression of COLGALT1 is related to normal or tumor, male or female, different grades or stages (Fig. 2A–D). Based on immunohistochemical images from the HPA database, we noticed tumor tissues have a greater level of COLGALT1 protein expression than normal tissues (Fig. 2E, F). In addition, we compared the expression of COLGALT1 in TCGA–KIRC samples with different clinicopathological characteristics. From TCGA, patients with KIRC are grouped based on their clinical characteristics, including race, age, stage, grade, gender, T, M, and N. The connections between COLGALT1 expression and these clinicopathological characteristics are illustrated in Fig. 3A–H. It suggested that the COLGALT1 is up-regulated in advanced tumors, regardless of their grade, stage, T, M and N, compared with early tumors (all *P* < 0.01). However, there was no significance between COLGALT1 expression and age, gender, race.

### Univariate and multivariate Cox regressions analysis

**Fig. 4:**
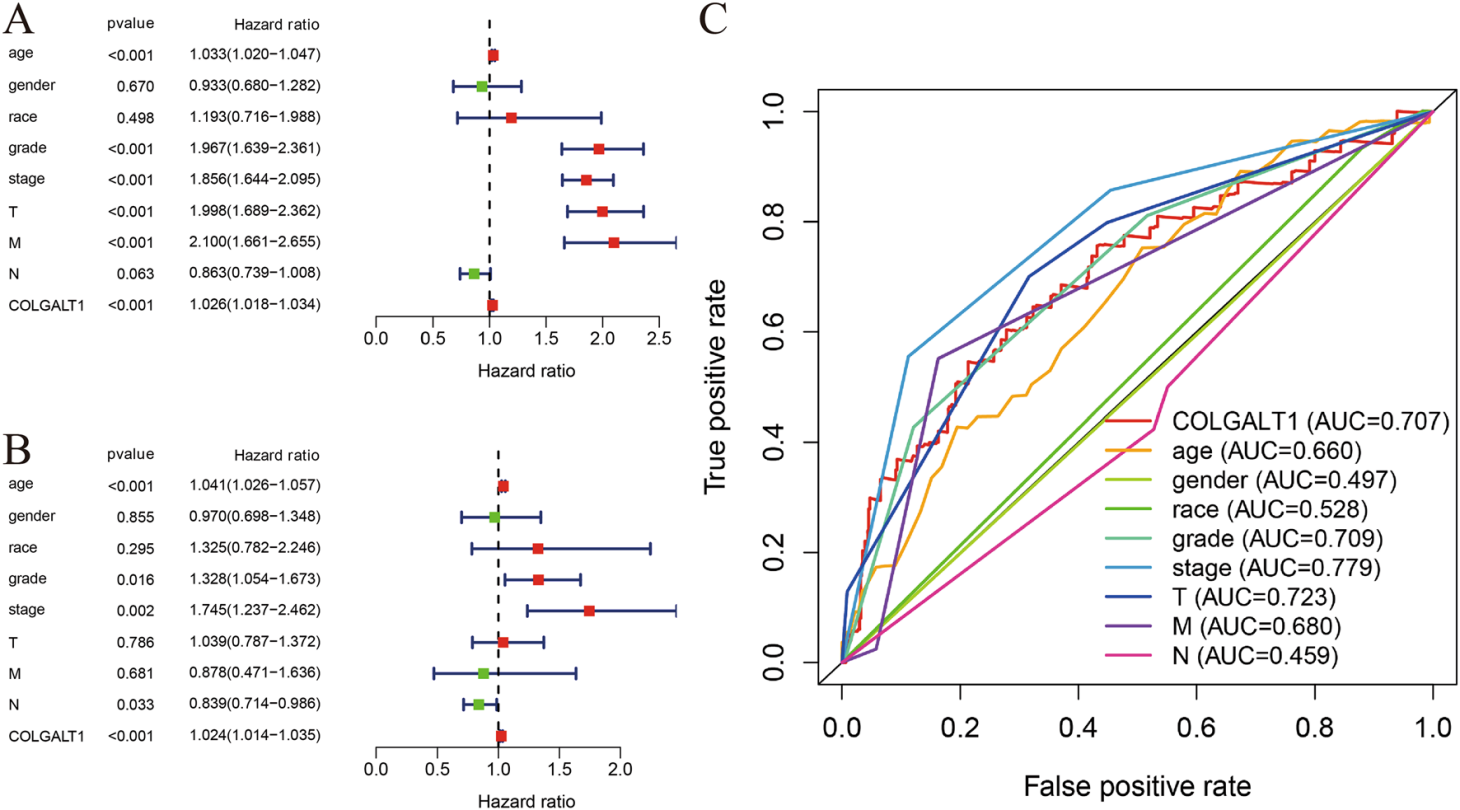
**A** Univariate cox regression analysis; **B** multivariate cox regression analysis; **C** multi-ROC curves of COLGALT1 and clinicopathologic characteristics

We evaluated clinical factors associated with OS using univariate and multivariate Cox regression analysis (Table 1). In the univariate Cox analysis, there was a strong relationship between the OS of KIRC patients and the COLGALT1 expression, as well as the pathological stage, grade, T stage, and distant metastasis (Fig. 4A). Multivariate Cox regression analysis showed that KIRC patients who had high levels of COLGALT1 expression had poor OS (HR = 1.024; *p* < 0.01). In addition, there were other clinicopathologic parameters, such as advanced stage and age correlated with poor overall survival (Fig. 4B). Overall, the index of COLGALT1 expression could be used as an independent prognostic indicator for KIRC. Analysis of the ROC curve showed that COLGALT1 expression and other clinicopathologic variables are prognostic indicators. The AUC for COLGALT1 expression (AUC = 0.707) was significantly higher than that of most clinical factors, such as lymph nodes status (0.459), gender (0.497), race (0.528), age (0.660) and M stage (0.680). Moreover, the AUC of COLGALT1 was merely less than T stage (0.723), grade (0.709) and pathological stage (0.779) (Fig. 4C).

**Table 1:** Prediction of overall survival using univariate and multivariate analyses of COLGALT expression level and clinicopathological variables

Variable	Univariate analysis			Multivariate analysis		
	HR	95% CI	*p*	HR	95% CI	*p*
Overall survival						
Age (year)	1.033	1.020–1.047	** < 0.001**	1.041	1.026–1.057	** < 0.001**
Gender	0.933	0.680–1.282	0.670	0.970	0.698–1.348	0.855
Race	1.193	0.716–1.988	0.498	1.325	0.782–2.246	0.295
Grade	1.967	1.639–2.361	** < 0.001**	1.328	1.054–1.673	0.016
Stage	1.856	1.644–2.095	** < 0.001**	1.745	1.237–2.462	**0.002**
T	1.998	1.689–2.362	** < 0.001**	1.039	0.787–1.372	0.786
M	2.100	1.661–2.655	** < 0.001**	0.878	0.471–1.636	0.681
N	0.863	0.739–1.008	0.063	0.839	0.714–0.986	0.033
COLGALT	1.026	1.018–1.034	** < 0.001**	1.024	1.014–1.035	** < 0.001**

Bold indicates* p* < 0.05

HR; Multivariate models were adjusted for age, grade, race, stage and T,N,M classification

*HR* hazard ratio, estimated from Cox proportional hazard regression model, *CI* confidence interval of the estimated

### GSEA identifies COLGALT1-related signaling pathways

To investigate potential signaling pathways in KIRC patients with high COLGALT1 expression, GSEA was performed [20]. According to |normalized enrichment score (NES)|> 1.5 and *p* value < 0.05, several significantly enriched signaling pathways were selected (Additional file 1: Figure S1 and Table 2). The result indicated that signaling pathways including Chemokine, Jak Stat, Nod-like receptor, Notch, P53, PPAR, Oxidative phosphorylation are differentially enriched in high or low COLGALT1 expression phenotype.

**Table 2:** Gene sets enrichment analysis of COLGALT mRNA expression in the ccRCC cohort

Gene set name	NES	NOM *p* value	FDR *q* value
KEGG_CHEMOKINE_SIGNALING_PATHWAY	−2.256	< 0.001	0.003
KEGG_JAK_STAT_SIGNALING_PATHWAY	−2.130	0.002	0.007
KEGG_NOD_LIKE_RECEPTOR_SIGNALING_PATHWAY	−2.204	0.002	0.005
KEGG_NOTCH_SIGNALING_PATHWAY	−2.089	< 0.001	0.007
KEGG_P53_SIGNALING_PATHWAY	−2.112	0.002	0.007
KEGG_PPAR_SIGNALING_PATHWAY	1.778	0.014	0.057
KEGG_OXIDATIVE_PHOSPHORYLATION	1.892	0.014	0.031

*NES* normalized enrichment score, *NOM* nominal, *FDR* false discovery rate

### The evaluation of PPI network, MSI, TMB, Neoantigen with the COLGALT1 expression in KIRC

**Fig. 5:**
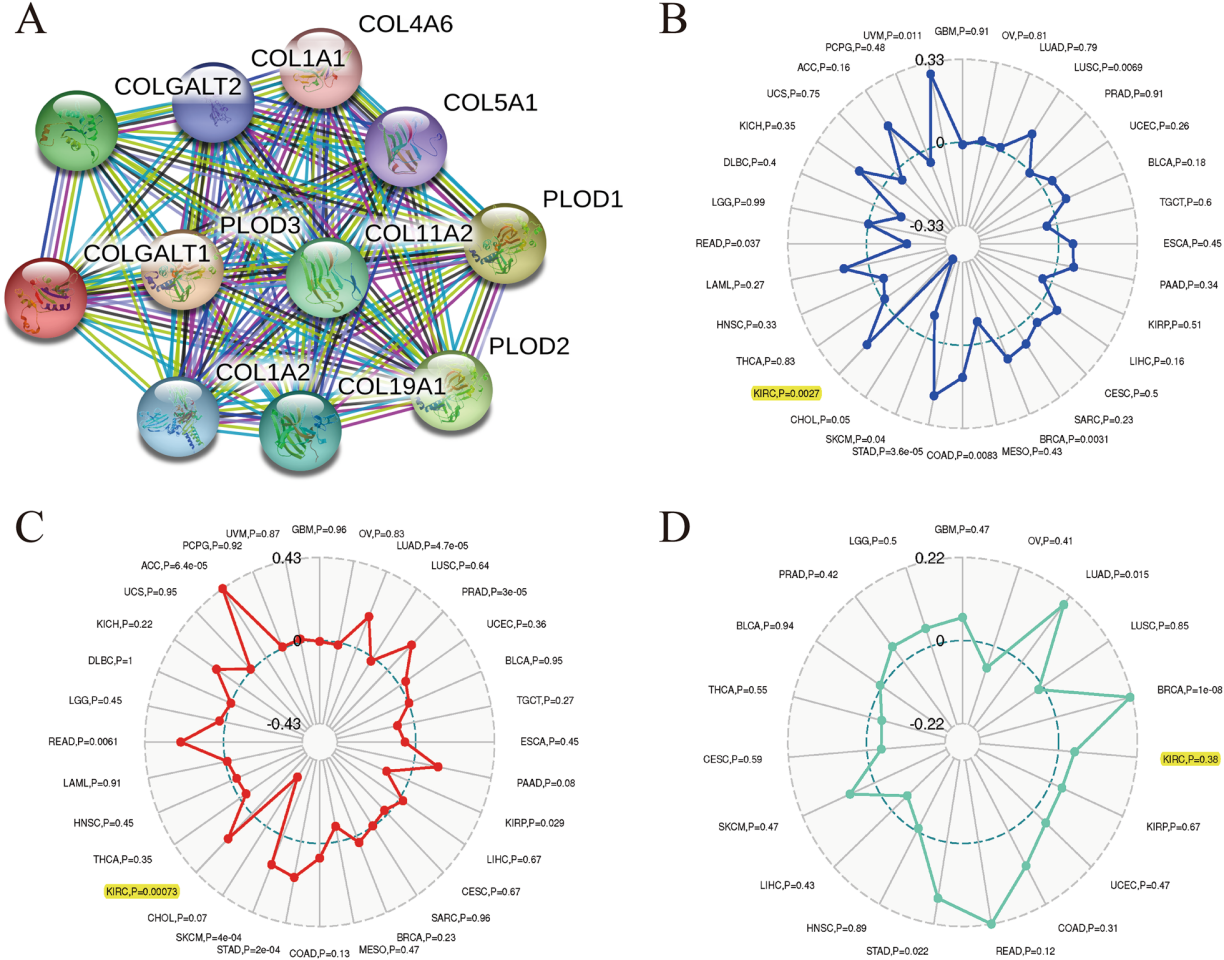
**A** PPI network analysis; the evaluation of **B** MSI, **C** TMB, **D** Neoantigen and COLGALT1 expression in TCGA–KIRC data set

STRING website tool was used to analyze the PPI network and the result revealed ten proteins most interacted to COLGALT1 (Fig. 5A). Neoantigens, microsatellite instability as well as tumor mutation burden has been found valid biomarkers for predicting prognostic and immune therapy response in a variety of tumors. According to the pan-cancer analysis of COLGALT1 and MSI, the differential expression of COLGALT1 is associated with MSI in STAD (p = 3.6e-05), COAD (*p* = 0.0083), BRCA (*p* = 0.0031), LUSC (*p* = 0.0069) and KIRC (*p* = 0.0027) (Fig. 5B). The correlation analysis of COLGALT1 and TMB indicated the expression of COLGALT1 is positively related to TMB in SKCM (*p* = 4e-04), STAD (*p* = 2e-04), PRAD (*p* = 3e-05), LUAD (*p* = 4.7e-05), ACCP (*p* = 6.4e-05), READ (*p* = 0.0061) and KIRC (*p* = 0.00073) (Fig. 5C). However, the association between COLGALT and Neoantigens was not statistically significant in TCGA–KIRC (*p* = 0.38) (Fig. 5D).

### Immunological features of COLGALT1 in TCGA–KIRC data set

**Fig. 6:**
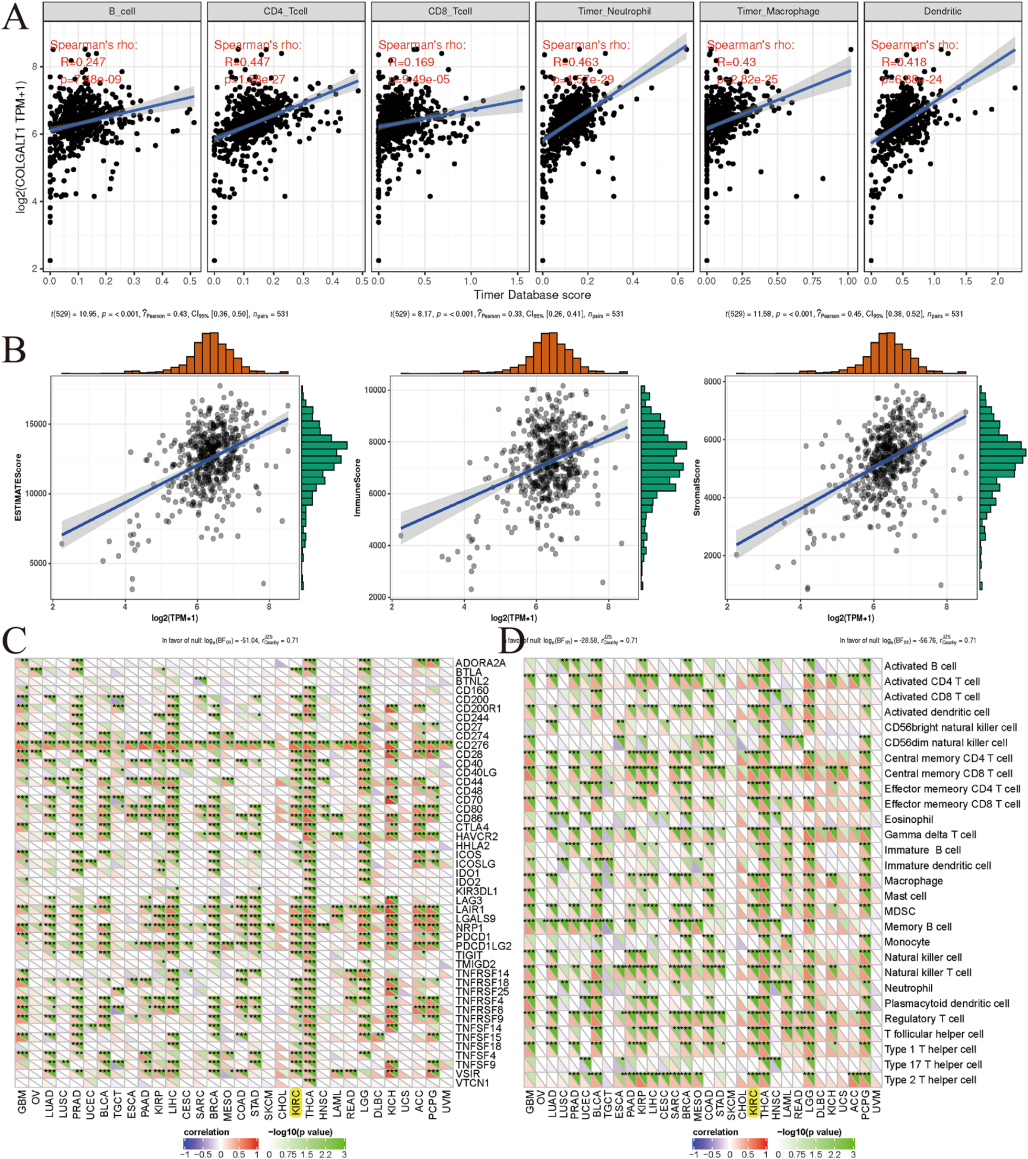
Correlation analysis of COLGALT1 expression and **A** immune cells infiltration; **B** tumour microenvironment; **C** immune checkpoint genes; **D** immune cells; *P < 0.05; **P < 0 .01; ***P < 0 .001

Through the website tool of TIMER, we investigated the association of COLGALT1 and various types of immune cell infiltration (B cells, dendritic cells, neutrophils, CD8+T cells, CD4+T cells and macrophages) in KIRC. The result suggested that the expression of COLGALT1 is positively associated with the infiltration of CD4+T cells (*p* = 1.88e-27), B cells (*p* = 7.48e-09), CD8+T cells (*p* = 9.49e-05), macrophages (*p* = 2.82e-25), neutrophils (*p* = 1.57e-29) and dendritic cells (*p* = 6.88e-24) in TCGA–KIRC data set (Fig. 6A). Furthermore, we employed another online tools of Sangerbox website to assess the relationships between COLGALT1 expression and tumor microenvironment in TCGA–KIRC data set. The results indicated the expression of COLGALT1 is positively correlated with the Stromal score, Immune score and Estimate score with a p value less than 0.001 (Fig. 6B). To study the potential targets of KIRC immunotherapy, the KIRC's mRNA sequencing data was utilized to assess the association between COLGALT1 and the acknowledged immune checkpoint genes. It suggested that COLGALT1 expression is strongly associated with relevant checkpoint genes such as BTLA, CD200, CD244, CD274, CD276, CD28, CD80 and so on (Fig. 6C), as well as immune cells such as activated CD4 T cell, effector memory CD4 T cell, central memory CD4 T cell, activated dendritic cell and so on in TCGA–KIRC data set (*P *< 0.05) (Fig. 6D).

### Prediction of immune response of COLGALT1 to immunotherapy in TCGA–KIRC data set

**Fig. 7:**
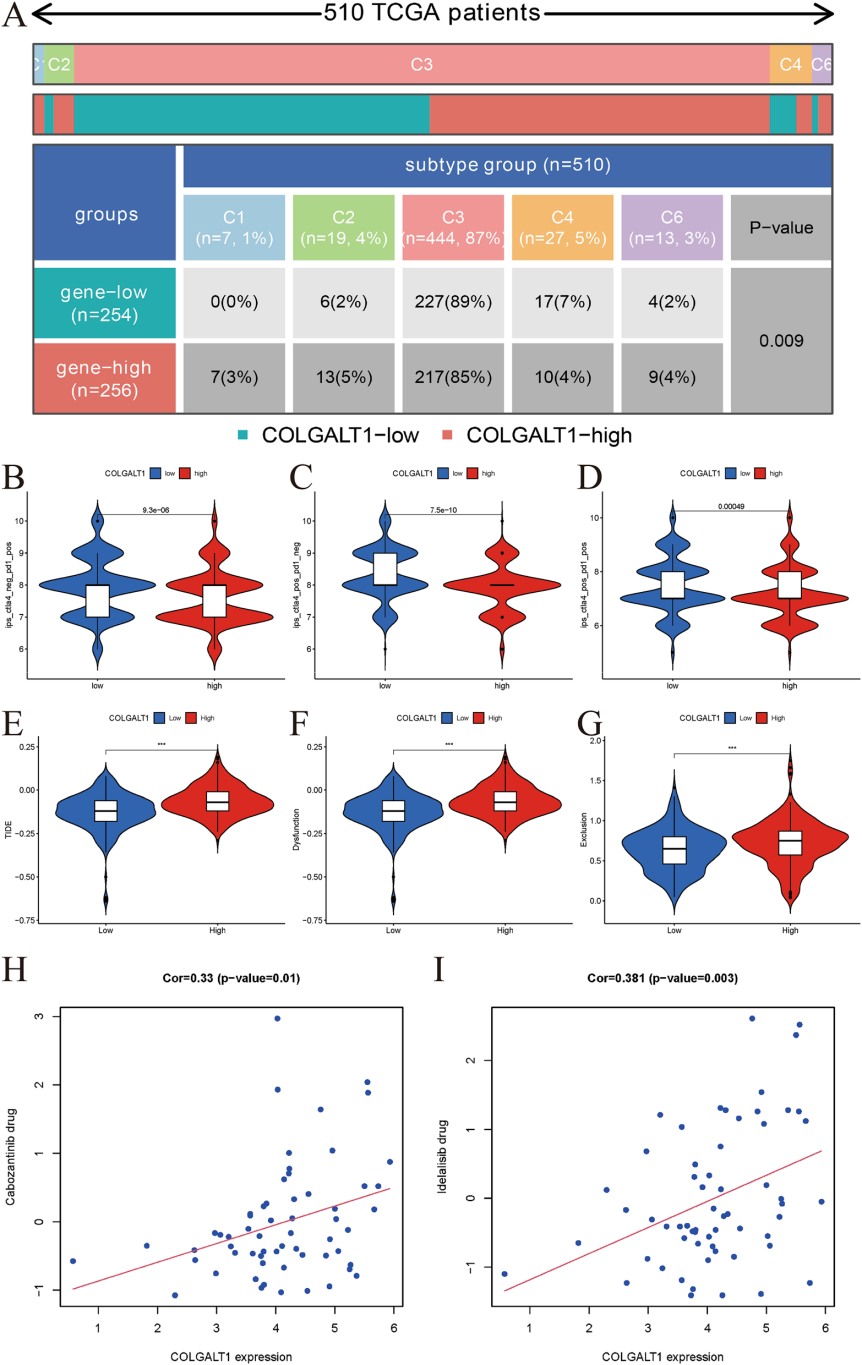
**A** Distributions of COLGALT1 expression in five types of immune subtypes; TCIA database was utilized to predict the immune response of COLGALT1 to **B** PD1 immunotherapy; **C** CTLA4 immunotherapy; **D** PD1 and CTLA4 immunotherapies; TIDE results of **E** TIDE score; **F** T cell dysfunction score; **G** T cell exclusion score in different COLGALT1 subgroups; **H**, **I** Correlations between sensitivity of chemotherapy drugs with COLGALT1 expression in CellMiner database

So far, six immune subtypes (ISs; C1–C6) of solid tumors have been identified according to the global transcriptome immune classification [21]. Figure 7A displays the distributions of COLGALT1 expression in five types of ISs, predominantly including C1 wound healing (*n* = 7, 1%), C6 TGF-beta dominant (*n* = 13, 3%) (*p* < 0.001), C4 lymphocyte depleted (*n* = 27, 5%), C3 inflammatory (*n* = 444, 87%), and C2 IFN-gamma dominant (*n* = 19, 4%). TCIA database was utilized to predict the immune response of COLGALT1 to CTLA4 or PD1 immunotherapies. Our results indicated that COLGALT1 was sensitive to both the CTLA4 and PD1 immunotherapies (all *P* < 0.001; Fig. 7B–D). TIDE was used to investigate whether immunotherapy could improve clinical outcomes for different COLGALT1 subgroups. Based on our results, the COLGALT1–low subgroup had a lower TIDE score than COLGALT1–high subgroup (Fig. 7E). In addition, we found that the COLGALT1–low subgroup had a lower T cell exclusion score and T cell dysfunction (Fig. 7F, G). Moreover, the sensitivity of anticancer drugs based on the COLGALT1 expression was performed through the CellMiner database. We found that the expression of COLGALT1 was significantly positively correlated with the sensitivity of cabozantinib and idelalisib drugs (both *p* ≤ 0.01) (Fig. 7H, I).

### Prediction of potential LncRNA/miRNA/COLGALT1 mRNA network in TCGA–KIRC data set

**Fig. 8:**
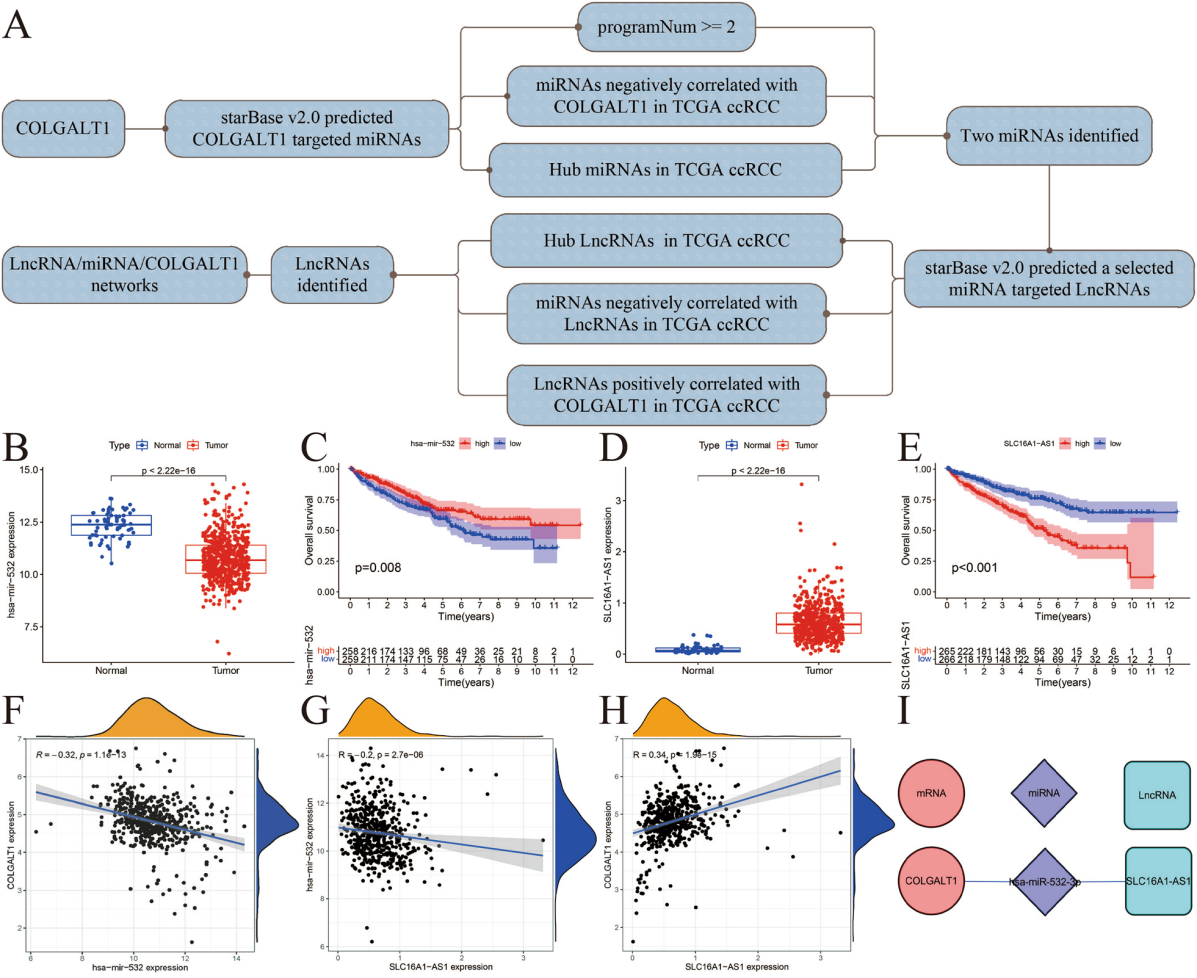
**A** Flowchart for prediction of LncRNA/miRNA/COLGALT1 mRNA network in TCGA–KIRC data set. **B** Expression of hsa-mir-502 in KIRC samples and normal samples. **C** Overall survival curve of hsa-mir-502 in KIRC. **D** Expression of SLC16A1-AS1 in KIRC samples and normal samples. **E** Overall survival curve of SLC16A1-AS1in KIRC. **F** Expression of hsa-mir-502 showed negative correlation with COLGALT1 expression in KIRC. **G** Expression of SLC16A1-AS1 showed negative correlation with SLC16A1-AS1 expression in KIRC. **H** Expression of SLC16A1-AS1 showed positive correlation with COLGALT1 expression in KIRC; **I** potential ceRNA network of SLC16A1-AS1/hsa-mir-502-3p/COLGALT1 in TCGA–KIRC data set

We carried out several analyses to find hub miRNA and LncRNA in TCGA–KIRC data set by starBase v2.0 website. We conducted further screening with the following strategies: (1) obtained miRNA was negatively correlated with COLGALT1; (2) obtained LncRNA was positively correlated with COLGALT1; (3) obtained miRNA was negatively correlated with LncRNA for further screening. Then, we used starBase v2.0 (for decoding the Interaction Networks of mRNAs and lncRNAs, miRNAs etc.) [22] to predict the LncRNA/miRNA/COLGALT1 network acting on KIRC. We ultimately found one potential LncRNA/miRNA/COLGALT1 network in KIRC (Fig. 8A). Furthermore, we conducted further analysis to verify above results. We found hsa-mir-502 is down-regulated in tumor tissues than normal tissues (*p* < 2.22e-16) and patients with higher hsa-mir-502 expression had a better prognosis (*p* = 0.008) (Fig. 8B–C). In addition, we discovered SLC16A1-AS1 is up-regulated in tumor tissues than normal tissues (*p* < 2.22e-16) and patients with low-SLC16A1-AS1 expression had a better prognosis than patients with high-SLC16A1-AS1 expression (*p* < 0.001) (Fig. 8D, E). We then carried out Pearson’s correlation analysis to investigate the relation between COLGALT1, hsa-mir-502 and SLC16A1-AS1 based on TCGA data set. As expected, we found hsa-mir-502 is negatively correlated to COLGALT1 (*R* = −0.32, *p* = 1.1e-13), SLC16A1-AS1 is negatively correlated to hsa-mir-502 (*R* = −0.2, *p* = 2.7e-06) and SLC16A1-AS1 is positively correlated to COLGALT1 (*R* = 0.34, *p* = 1.9e-15) (Fig. 8F–H). Overall, we predicted the potential ceRNA network of SLC16A1-AS1/hsa-mir-502-3p/COLGALT1 in TCGA–KIRC data set (Fig. 8I).

### Evaluating the expression and function of COLGALT1 in vitro

**Fig. 9:**
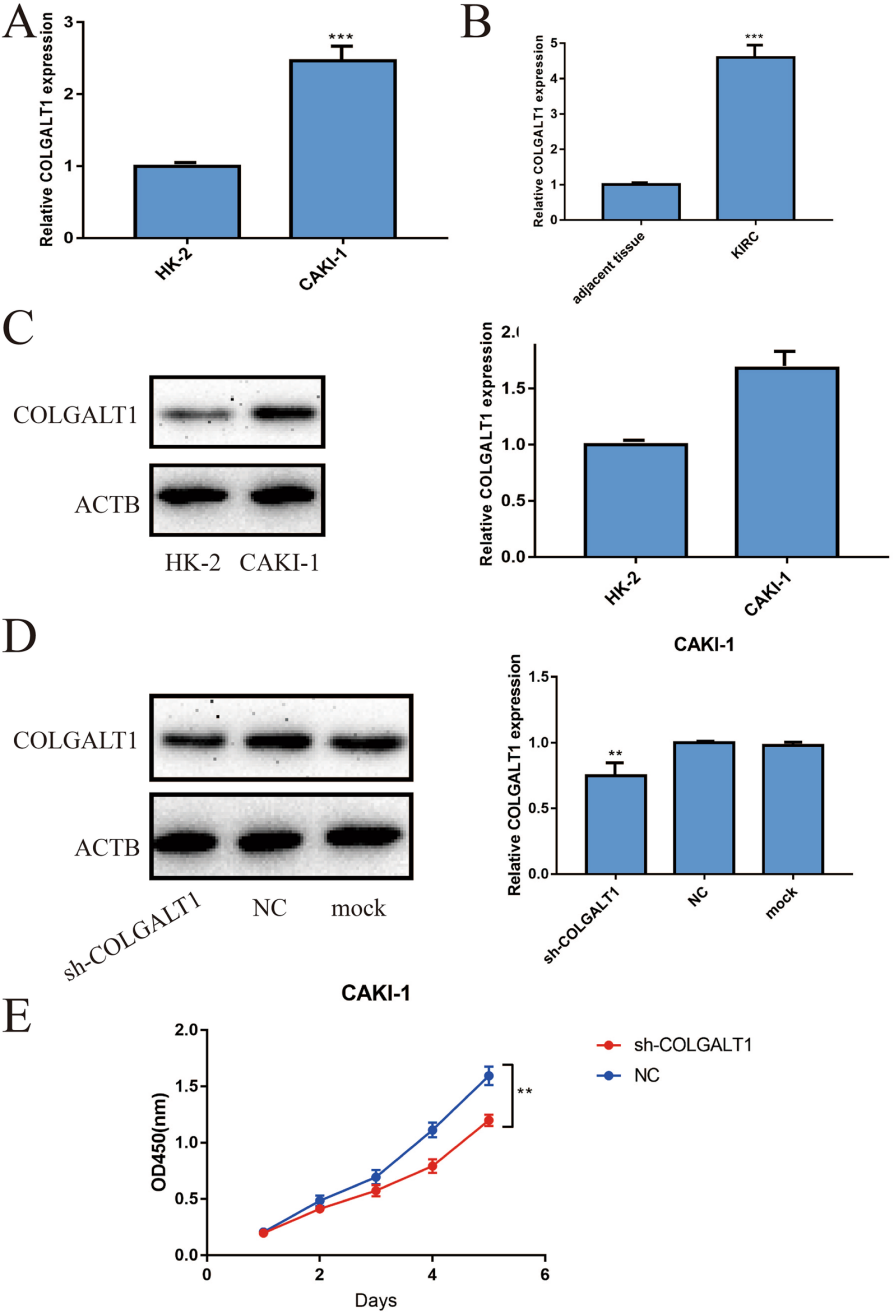
**A** qRT-PCR results indicated that COLGALT1 mRNA expression is higher in KIRC cell line Caki-1 compared with normal HK-2 cells; **B** qRT-PCR results indicated that COLGALT1 mRNA expression is higher in KIRC tissues compared with adjacent normal kidney tissues; **C** western blot results indicated that COLGALT1 protein expression is higher in KIRC cell line Caki-1 compared with normal HK-2 cells and its bar graph; **D** western blot results indicated that the COLGALT1 protein expression in KIRC cell lines CAKI-1 transfected with COLGALT1 knockdown vector and negative control vector and its bar graph. **E** CCK-8 experiments of CAKI-1 transfected with COLGALT1 knockdown vector and negative control vector

The result was similar with previous results based on TCGA database. Through qRT-PCR experiment, we verified the COLGALT1 mRNA expression which is up-regulated in the KIRC cell line CAKI-1 compared with the renal tubular epithelial cell HK-2 (Fig. 9A). Meanwhile, we found the mRNA of COLGALT1 is undoubtedly up-regulated in KIRC samples compared with the adjacent tissues collected in our institution (Fig. 9B). Next, we assessed the protein expression of COLGALT1 in CAKI-1 and HK-2 through Western Blot. The result revealed the COLGALT1 protein expression is highly expressed in CAKI-1 compared with HK-2 (Fig. 9C). Furthermore, we used the COLGALT1 knockdown vector to investigate the potential function of COLGALT1 in KIRC cell line CAKI-1. We verified the transfection efficiency by Western Blot and the relative expression of COLGALT1 was visualized through the GraphPad Prism software (Fig. 9D). In addition, CCK-8 experiment was conducted and we found that COLGALT1 was significantly related to cell proliferation (Fig. 9E).

## Discussion

KIRC was commonly found to be characterized with the post translational modifications which regulate the initiation and progression of various malignant tumour [23]. Protein glycosylation, as a post-translational modification has been proved closely related to the development and progression of cancer [24]. Collagen triple helix formation is facilitated by COLGALT1, which contribute to the biosynthesis of collagen type IV by acting on collagen glycosylation [10, 25]. We found that COLGALT1 is up-regulated in KIRC. However, so far there have been no studies investigating the role of COLGALT1 in the prognosis and potential therapeutic value in KIRC.

We researched the expression of COLGALT1 between KIRC tissues and normal kidney tissues from public databases. The results indicated that COLGALT1 was significantly up-regulated in KIRC tissues than corresponding normal tissues regardless of mRNA or protein level. In addition, we noticed that high expression of COLGALT1 mRNA or protein level is positively correlated with advanced or higher malignancies in KIRC. By reviewing existing literature, we found another gene COLGALT2 which also initiated collagen glycosylation was up-regulated in metastatic osteosarcoma tissues than primary osteosarcoma tissues [26]. To assess the prognosis value of COLGALT1 in KIRC, we carried out the cox regression analysis and survival analysis with clinical information and sequencing data obtained from public databases. In addition, in KIRC patients, high expression of COLGALT1 often predicted a poor prognosis. Furthermore, we verified the expression of COLGALT1 in KIRC tissues and adjacent normal tissues collected from clinical institution through PCR. The results indicated COLGALT1 is up-regulated in KIRC tissues compared with adjacent normal tissues. Then, according to the result of western blot, we found similar expression at the protein level of COLGALT1 in both cells and tissues. These results are consistent with previous outcome derived from bioinformatics analysis. Moreover, we transfected COLGALT1 knockdown vector and negative control vector to evaluate the function of COLGALT1 in KIRC cell lines. It suggested that knockdown of COLGALT1 obviously inhibited the cell proliferation and could induce the cell death. We noticed another type of collagen galactosyltransferase Lysyl Hydroxylase (LH) and glycosyltransferase-deficient mutants or targeted destruction of LH3 in cells result in abnormal cell morphology and cell death [27]. This indicated the significance of glycosyltransferase activity for cell growth and viability, and abnormal glycosylation could cause the initiation, progression of tumor. Therefore, we speculated that the occurrence and progression of tumors might be closely related to excessive or abnormal glycosylation caused by the up-regulated COLGALT1 and further study was still needed to verify the related mechanism of COLGALT1 in KIRC.

GSEA was carried out further and we noticed COLGALT1 might play an important role in KIRC through the following signaling pathways including the Chemokine pathway, PPAR pathway, Nod-like receptor pathway, Notch pathway, P53 pathway, Jak Stat pathway and Oxidative phosphorylation pathway. We also found the relationships between COLGALT1 and ten of the most relevant genes through the PPI network. Further experiments in vitro were needed to confirm the potential relationships between them.

Next, several correlation analyses were processed to investigate the value of COLGALT1 for the immunotherapy of KIRC, and we noticed the correlation between COLGALT1 and related immune features including TMB and MSI. In recent years, TMB and MSI haD become emerging biomarkers for predicting response to Immune Checkpoint Inhibitor (ICI) treatment [28, 29]. It meant the potential ICI therapy of COLGALT1 for KIRC. Meanwhile, correlation analysis indicated the COLGALT1 expression might be connected with related immune cells pathway-like central memory CD4 T cell, activated CD4 T cell, activated dendritic cell and effector memory CD4 T cell. The correlation analysis of COLGALT1 and immune checkpoint genes indicated the COLGALT1 expression might be connected with checkpoint genes such as BTLA, CD200, CD244, CD274, CD276, CD28 and CD80. So far, there was still a lack of immunotherapy research on related checkpoint genes and immune pathways in KIRC as well as the investigation between COLGALT1 and related immunologically targets. All these results provided a new perspective of COLGALT1 for the immunotherapy of KIRC and revealed the potential targets and related immune pathways. Therefore, differential expression of COLGALT1 might contribute to tumor immunotherapy.

We divided KIRC samples into a COLGALT1–high subgroup and COLGALT1–low subgroup from TCGA database. In addition, we observed the immunophenotyping composition of these groups with identified six immune subtypes (ISs) (C1–C6) according to the global transcriptome immune classification [21], we found 5 ISs including C3 inflammatory, C2 IFN-gamma dominant, C1 wound healing, C6 TGF-beta dominant, and C4 lymphocyte depleted. COLGALT1 was found to be differently distributed in these immune subtypes. In the immune circulatory system, immune checkpoints such as PD1 and CTLA-4 have inhibitory effects on T cell function, which could help tumors resist apoptosis caused by immune responses and promote tumor progression. In recent years, anti-PD1 and anti-CTLA-4 immunotherapy had made good progress in the treatment of tumors [30, 31]. Intriguingly, TCIA analysis revealed that COLGALT1 expression varied depending on the ICI treatment (anti-PD1 and anti-CTLA4). Moreover, TIDE is a computational method used to identify factors contributing to two mechanisms of tumor immune escape: increasing T cell dysfunction in tumors with high cytotoxic T lymphocyte infiltration (CTLs) and preventing T cell infiltration in tumors with low CTL levels [32]. The TIDE prediction score correlated with T cell dysfunction and T cell exclusion in COLGALT1–high tumors and thus describes two different immune escape mechanisms. In this study, we found patients in COLGALT1–high subgroup had higher TIDE, T cell dysfunction score and T cell exclusion score, so the reason for their lower ICI response might be that their T cells had been excluded and dysfunctional. It suggested that patients in COLGALT1–low subgroup might benefit more from Immune checkpoint inhibitor therapy than COLGALT1–high patients. In addition, higher TIDE predictions were correlated with poorer results. Therefore, patients in COLGALT1–high subgroup with high TIDE score might mean a worse prognosis than that in COLGALT1–low subgroup. It was consistent with our initial survival analysis. Furthermore, we used CellMiner database to explore potential Chemotherapy drugs that were sensitive to patients with high expression of COLGALT1 in KIRC [33]. We found the expression of COLGALT1 was significantly positively associated with the sensitivity of cabozantinib and idelalisib drugs. It suggested patients with high expression of COLGALT1 could benefit from these drugs.

We further explored the potential LncRNA/miRNA/COLGALT1 network which could work in KIRC. Based on the TCGA database, we had successfully discovered a network SLC16A1-AS1/hsa-mir-502-3p/COLGALT1 that might act on KIRC through a series of analysis. The expression characteristics were followed: high expression of SLC16A1-AS1, high COLGALT1 expression, and low hsa-mir-502 expression in KIRC. In the literature, it had been reported that genetic changes in miR-502 could reduce the risk of breast cancer [34]. In colon cancer, expression miR-502-5p might be closely associated to the occurrence of colon cancer through cell glycolysis, invasion, migration, and proliferation pathways [35]. Gastric cancer cells were stimulated to proliferate and metastasize by CircDLST through sponging miR-502-5p, which activated the NRAS/MEK1/ERK1/2 signaling cascade [36]. In bladder cancer, lncRNA-SLC16A1-AS1 promoted tumor invasion by participating in the formation of a hybrid oxidative phosphorylation/glycolysis cell phenotype [37]. Oral squamous cell carcinoma (OSCC) samples contained high levels of SLC16A1-AS1 expression compared to normal tissue samples [38]. In summary, we found that high SLC16A1-AS1 expression and the low hsa-mir-502 expression were concerned with the occurrence and invasion of various tumors. The ceRNA network of SLC16A1-AS1/hsa-mir-502-3p/COLGALT1 still needed further molecular biology experiments to verify the possible mechanism in KIRC.

The advantages of COLGALT1 compared with other KIRC prognostic markers should also be discussed. As for its diagnostic efficiency, the AUC for COLGALT1 expression (AUC = 0.707) was significantly higher than that of most clinical factors, such as lymph nodes status (0.459), gender (0.497), race (0.528), age (0.660) and M stage (0.680). Moreover, the AUC of COLGALT1 was merely less than T stage (0.723), grade (0.709) and pathological stage (0.779). In terms of its prognostic effects, COLGALT1 could serve as an independent prognostic biomarker for KIRC. Moreover, we found that COLGALT1 was significantly associated with immunity and could predict immune responses of immunotherapy by means of TIDE and TCIA. Last but not least, the SLC16A1-AS1/hsa-mir-502-3p/COLGALT1 mRNA axis was revealed by us for its potential mechanisms. Taken together, we believed that COLGALT1 could be a potential biomarker to investigate the mechanism and clinical therapeutic value of KIRC.

## Conclusions

Our current study indicated COLGALT1 was up-regulated in KIRC samples. COLGALT1 was found to be a reliable prognostic factor for KIRC in univariate/multivariate analysis of cox regression, and high expression of COLGALT1 meant poor prognosis. Experiments in vitro suggested that COLGALT1 was up-regulated and related to cell proliferation significantly in KIRC. By means of a series of bioinformatics analysis techniques, we found COLGALT1 might strongly correlate with immunity which could act as a valid immune-related prognostic indicator for KIRC. In addition, we predicted high expression of COLGALT1 could benefit from cabozantinib and idelalisib drugs. We even identified a ceRNA network of SLC16A1-AS1/hsa-mir-502-3p/COLGALT1 for KIRC. More evidence and further experiments were still needed to verify our findings.

## Supplementary Information

**Additional file 1: Figure S1. **Gene Set Enrichment Analysis of COLGALT1 in TCGA–KIRC data set. (A) Chemokine pathway; (B) Jak Stat pathway; (C) Nod-like receptor pathway; (D) Notch pathway; (E) Oxidative phosphorylation pathway; (F) P53 pathway; (G) PPAR pathway; (H) Summarizing of these signalling pathways.

## Data Availability

All the data used to support the findings of this study are included within the article. Please contact author for data requests.

## Abbreviations

COLGALT1: Collagen beta(1-O)galactosyltransferase 1
KIRC: Kidney renal clear cell carcinoma
ceRNA: Competing endogenous RNA
OS: Overall survival
MSI: Microsatellite instability
TMB: Tumor mutational burden
RCC: Renal cell carcinoma
PTM: Post-translational modification
DEGs: Different expression genes
ICGC: International Cancer Genome Consortium
GEO: Gene Expression Omnibus
CPTAC: Clinical proteomic tumor analysis consortium
HPA: Human Protein Atlas
CCK-8: Cell Counting Kit-8
KEGG: Kyoto Encyclopedia of Genes and Genomes
TIDE: Tumor Immune Dysfunction and Exclusion
GSEA: Gene set enrichment analysis
